# Experiences of the Postoperative Recovery Process: An Interview Study

**DOI:** 10.2174/1874434600802010001

**Published:** 2008-01-04

**Authors:** R Allvin, M Ehnfors, N Rawal, E Idvall

**Affiliations:** 1 Department of Anesthesiology and Intensive Care, Orebro University Hospital, Sweden; 2 Department of Clinical Medicine, Orebro University, Sweden; 3 Centre for Evidence Based Medicine and Assessment of Medical Technology, Orebro, Sweden; 4 Department of Health Sciences, Orebro University, Sweden; 5 Research Section, Kalmar County Council, Sweden; 6 Department of Medicine and Health, Linkoping University, Sweden

**Keywords:** Postoperative, recovery, experience, interview, content analysis.

## Abstract

Few researchers have described postoperative recovery from a broad, overall perspective. In this article the authors describe a study focusing on patient and staff experiences of postoperative recovery using a qualitative descriptive design to obtain a description of the phenomenon. They performed 10 individual interviews with patients who had undergone abdominal or gynecological surgery and 7 group interviews with registered nurses working on surgical and gynecological wards and in primary care centers, surgeons from surgical and gynecological departments, and in-patients from a gynecological ward. The authors analyzed data using qualitative content analysis. Postoperative recovery is described as a Dynamic Process in an Endeavour to Continue With Everyday Life. This theme was further highlighted by the categories Experiences of the core of recovery and Experiences of factors influencing recovery. Knowledge from this study will help caregivers support patients during their recovery from surgery.

## INTRODUCTION

Throughout the world millions of patients undergo surgery each year. Increased knowledge, the development of anesthesiology and surgery techniques, and economic pressures has all resulted in shortened hospitalization time [1, 2]. For example, it has been proposed that even older patients can be discharged from hospital within 2 or 3 days after major colonic surgery [3]. As a result of this trend, much of the responsibility for postoperative recovery is being shifted to the patients and their families. This shift has probably been carried out without considering patients’ possibility or ability to take care of their own recovery. Therefore, it is important to increase our knowledge and understanding of postoperative recovery.

Postoperative recovery following a variety of surgical procedures has been studied [4, 5]. In general, researchers have evaluated short-term recovery by studying length of hospitalization [6] and return to normal life, focusing on such symptoms as pain [7, 8], postoperative nausea and vomiting [9], sleep disturbance [10], fatigue [11], functional level [12], and cognitive dysfunction [13]. This research line has been directed mainly toward a single symptom [14, 15] and has increased our knowledge of isolated postoperative symptoms; however, little is known about other areas of postoperative recovery.

Emphasis in qualitative studies has been on patient experiences in postoperative recovery, primarily factors associated with specific diseases. In a study of patient experiences after gastrointestinal cancer surgery Olsson and colleagues [16] described recovery as a mixture of feelings between hope and doubt, and a will to break free from the consequences of the disease and treatment. A study of men’s experiences during recovery after radical prostatectomy demonstrated that adjustments in the weeks after discharge were the worst aspect of the prostatectomy experience [17]. This was reported to be due to the urinary catheter and its negative effects on the lives of these men. In general qualitative research has focused more on postoperative recovery as a part of patients` experiences of an illness than on recovery itself.

Despite the fact that postoperative recovery is a commonly used concept there is a need among clinicians working in the postoperative context to get a deeper insight of the course of events during recovery. Otherwise, there is a clear risk that a concept like postoperative recovery is being used without reflection. The rationale for the present study was to acquire increased knowledge and understanding of recovery after surgery. To do so, it seems important to take both the patients` unique, subjective experiences and the vast knowledge of health care professionals into account. Patients have highly personal and radically distinctive experiences of recovery, whereas many health care providers have considerable experience based on caring for a large number of individuals. Therefore, the aim of this study was to describe patient and staff experiences of patient recovery after surgery.

## METHODS AND PARTICIPANTS

In our study we employed a qualitative descriptive method as proposed by Sandelowski [18], using semi-structured interviews to obtain a description of postoperative recovery. To obtain data that vary widely, which was seen as necessary to capture both common and unique manifestations of postoperative recovery from different perspectives, we used purposeful sampling with maximum variation [18]. In all, 10 individual interviews and 7 focus group interviews (14 patients and 28 staff members) were performed. Ten patients (5 women and 5 men aged 34 to 76 years) who had undergone abdominal or gynecological surgery participated in individual interviews 3 weeks to 1 year post surgery. In addition to these interviews, we conducted seven focus group interviews, one with a group of in-patients from a gynecological ward (4 women aged 43 to 52 years), two with groups of registered nurses from surgical and gynecological wards (*n* = 6 + 6; 11 women and 1 man aged 27 to 52 years), two with groups of registered nurses from primary care in health care centers (*n* = 4 + 4; 8 women aged 39 to 61 years), and two with groups of surgeons from surgical and gynecological departments (*n* = 4 + 4; 5 women and 3 men aged 40 to 56 years).

We decided that to be included in the study, nurses had to have a minimum of 6 months` experience in surgical, gynecological, or primary health care and physicians had to be surgical or gynecological specialists. Nurses were selected for participation by their head nurse, whereas surgeons were selected by the head of their department at the hospital. Furthermore, the head nurse elected the patients for the group interviews. For the individual patient interviews, we used purposeful sampling to identify respondents according to type of surgery, time since surgery, and gender. Types of surgery were chosen based on the fact that they were considered to require a recovery period after discharge from hospital. Patient informants were to be capable of being interviewed, and both patient and staff informants were to participate voluntarily. Once the informants for the group interviews had been selected, the first author (I) contacted them and provided both verbal and written information. For the individual interviews I wrote a letter and then telephoned a week later and asked if they would be willing to participate, to which all agreed. After this I provided both verbal and written information. All informants were given the opportunity to ask questions.

### Data Collection

The study was carried out at a university hospital in central Sweden. Data were collected during late 2003 and 2004.

#### Individual Interviews

I conducted all interviews and asked the respondents to describe their experiences from the day of the surgery until the day of the interview. The main question was Tell me what it has been like to recover from surgery. I asked follow-up questions to develop or clarify the narrative further. All interviews were audiotaped in the respondent’s home (*n* = 4) or in a room at the hospital (*n* = 6), depending on the respondent’s preference. Interviews lasted between 25 and 60 minutes.

#### Focus Group Interviews

Focus group interviews are especially useful when researchers wish to explore a wide range of opinions, perceptions, and concerns that people have regarding a particular topic [18, 19]. This approach is useful as a data collection method on its own or in combination with other procedures, such as individual interviews [20, 21]. Time was provided at the start of each interview to clarify information. We used an interview guide with open-ended questions during the interviews so that the moderator could identify topics that had not been discussed and to direct participant dialogue to explore these topics. I was the moderator for all the focus group interviews, whereas the assistant moderators differed. I started the group discussion with the question Tell me what it is like for patients to recover from surgery. To help clarify the topic, I explored it further by repeatedly formulating similar questions from slightly different perspectives. The assistant moderators made notes and observed the group dynamics. After each interview we discussed our impressions and experiences. The interviews, which lasted between 60 and 90 minutes, were audiotaped in secluded rooms at the hospital.

### Ethical Considerations

This study was planned and implemented based on ethical principles commonly applied in clinical research. All respondents were given written information about the study and informed that participation was voluntary. All respondents gave their informed consent to enrolment in the study and were guaranteed confidentiality. The study was reviewed and approved by the research ethics committee at Orebro University (§ 104-204/03).

### Data Analysis

In the analysis we aimed to identify parts of the text relevant to providing a description of patient and staff experiences of postoperative recovery. Data were analyzed inductively using the principles of conventional qualitative content analysis described by Hsieh & Shannon [22], which was carried out in several steps [23].

The first step was to transcribe the interviews verbatim, which was done by the first author, who had also conducted all the interviews. To become even more familiar with the text and gain a sense of the whole, I read the text several times. Throughout these readings, I made notes on the transcript to document the first impression of what patients and staff were telling in their narratives, focusing on postoperative recovery.

The next step was to highlight the text that appeared to describe these experiences. These so-called meaning units were put together and constituted the units of analysis, still representing the verbatim words of patients and staff. I then condensed the meaning units into shorter expressions, which remained close to the text, but filtered out irrelevant information. I then coded the condensed meaning units separately at a manifest level of content. I compared the various codes and combined them with each other in an attempt to establish subcategories and categories for the purpose of describing different parts of the experiences of postoperative recovery. At this stage of the analytic process all of the subcategories and categories were put together in a diagram, focusing on recovery, to provide a sense of what the text was saying. The codes, subcategories, and categories were discussed by all authors and revised according to this discussion. Finally, the underlying meaning (i.e., the latent content) was formulated into a theme answering the research question. All authors read parts of the analyzed text, and we reached consensus on the analysis. We also compared the findings once more to the interview transcripts to identify quotations that best exemplified the findings. Throughout the analysis process described above, there was constant back-and-forth movement between different parts of the text.

## RESULTS

Patients and staff described postoperative recovery as A Dynamic Process in an Endeavor to Continue With Everyday Life. This constituted the overall theme based on two categories: Experiences of the core of recovery, with the subcategories unpleasant physical symptoms, disturbances in emotional well-being, a period of regaining functions, and a period of reestablishing activities; and Experiences of factors influencing recovery, with the subcategories antecedents to recovery, time to recover, support and encouragement, regular appropriate information, and setbacks during recovery. These findings are presented in the following text with representative quotations used to illustrate the findings.

### A Dynamic Process in an Endeavor to Continue With Everyday Life

The overall theme of postoperative recovery can be understood as a process extended over a time when individuals were striving and struggling to gain independence and return to everyday life. The essence of the recovery process was a desire to decrease unpleasant physical symptoms and reach a level of emotional well-being as well as regain functions and reestablish activities. The regaining of functions was more like a passive course of events that was out of the individual’s control, whereas reestablishing activities entailed conscious acts. Even if the postoperative recovery process extended over several months, it was still a limited period. In this context, the initial condition before surgery was considered as everyday life. This everyday life was interrupted by the surgical procedure and by the recovery process, which started immediately after surgery. The return to everyday life after the recovery process was not always a life that was comparable to life before the surgery.

Although it was easy to specify the start of the recovery process, it was more difficult to determine an achieved condition as the end, as the achieved condition could involve changes compared to the initial condition. The postoperative recovery process was influenced by diverse factors that could affect recovery in a positive or a negative direction. Our understanding of this process is further illustrated in Fig. (**1**).

### Experiences of the Core of Recovery

Experiences of unpleasant physical symptoms and disturbances in emotional well-being as well as a period of regaining different functions and a period of reestablishing activities were essential parts of the postoperative recovery process.

#### Unpleasant Physical Symptoms

A number of physical symptoms occurred after surgery. Pain and nausea were almost exclusively associated with the early recovery phase during hospitalization. These symptoms were experienced as uncomfortable and complicated the mobilization process. For those who experienced postoperative pain, effective pain treatment was felt to reduce the unpleasant sensation and made it possible to get out of bed and reestablish various activities. Some informants reflected on the fact that they did not experience pain and were convinced that it was due to the standard pain treatment provided. As well, constipation and diarrhea were problematic both during hospitalization and after discharge. These symptoms were often unexpected and experienced as extremely uncomfortable. A symptom occurring mainly after participants had returned to regular activities and work was fatigue, which was expressed as feelings of weakness and fragility with a lack of strength and energy.

“Many patients say that they do not understand where their strength has gone. We may experience them as recovered at discharge. However, when talking with them a few weeks later, some of them say that it took a long time to get their strength back. That is included in recovery”.

#### Disturbances in Emotional Well-Being

During hospitalization issues such as wound, drainage and different types of tubes caused anxiety and fear, all of which resulted in an unwillingness to participate in the mobilization process. Uncertainty about the future and anxiety about the diagnosis were reported regardless of whether the uncertainty or anxiety had its origin in a real event or whether it was a general anxiety that was caused by experiencing a serious illness. The major portion of the emotional reactions, however, appeared during a later phase, when the physical condition was under control. During the early recovery phase, a great deal of attention from family and friends was noted. However, this attention could decrease over time, even to the point where patients felt abandoned, which resulted in dispirited and disengaged feelings. These feelings were also reported by patients with various types of serious diseases. This emotional suffering represented an existential aspect of the postoperative recovery process.

“In the beginning, my illness was the main topic. Everyone I knew talked with me, and with each other about my situation. But after a while, it seemed that the only person who was concerned with my illness was me”.

#### A Period of Regaining Functions

Regaining different functions was described as a prerequisite to reestablishing preoperative activities. During hospitalization it mainly included basic functions such as urination, bowel function, and intake of fluid and food. Resuming normal bowel function was described as being surprisingly troublesome. During a later phase, the descriptions related more to regaining muscle strength to be able to manage exercising, lifting heavy things, and practicing outdoor activities. The regaining of functions took place over an extended period, which involved a successive weaning from dependency on support from others.

The prerequisites to regaining functions varied. For some individuals the recovery process led to improved functioning. “Orthopedic patients are very happy with their new hip joints. There is an obvious change in their life. This is what I call ´real recovery` - when you can see a significant difference in the patient’s life.” Other patients never fully recovered their preoperative functions.

Apart from physical functions, the importance of regaining social functions, such as returning to the role of parent, spouse, or workmate was emphasized. This was reported to occur later than the regaining of physical functions.

#### A Period of Reestablishing Activities

The reestablishment of activities had to be adjusted to fall in line with the patients` actual capacity. To initiate this process, the individual required strong self-discipline. During hospitalization such discipline mainly involved successive progress in the areas of mobilization, toileting, and personal hygiene. After hospital discharge, other activities were successively performed, starting with indoor exercise, before the patients gained the courage to leave the house. After a while the indoor exercise was supplemented with other activities (e.g., playing golf or tennis).

“When I returned home, I went for walks. In the beginning, I did not take long walks. I was always completely exhausted after my walks, but still every day I managed to increase the distance by a few meters”.

As with the regaining of functions, the reestablishment of activities took place over an extended period involving a successive weaning from dependency on the support of others.

### Experiences of Factors Influencing Recovery

Several factors were described as important in influencing recovery. Antecedents to recovery, the time required to recover, support and encouragement, regular appropriate information, and setbacks during recovery were all thought to affect the recovery process in positive or negative directions.

#### Antecedents to Recovery

Factors concerning the patient’s status or situation before surgery, such as age, preoperative physical status, diagnosis, and type of surgery, were thought to be important antecedents to recovery. Diagnosis and prognosis were believed to affect an individual’s desire to participate during recovery. In line with this notion, the demands and expectations placed on severely ill patients were not as high as they were for other patients. Recovery was also reported to vary depending on whether surgery was performed on an elective or acute basis. Furthermore, personal preoperative factors such as previous experience of postoperative recovery were noted to have either positive or negative influences. Positive experiences were viewed as helping individuals deal more effectively with the challenges of their next encounter with healthcare. At the same time, previous negative experiences of surgery were thought to transform into a positive experience if these negative experiences did not recur after the new surgery. Positive preoperative expectations about surgery outcome were also reported as important. “I looked forward to being rid of these bleedings, as they were extremely difficult to deal with”. In this context the importance of having realistic expectations was stressed.

#### Time to Recover

An important factor concerned the time necessary to recover, both in hospital and after discharge. The importance of being discharged when individuals felt ready to return home was stressed. “I think it is of utmost importance that you are ready for this decision [discharge].” During the late recovery phase the possibility of being on sick leave was described as a determining factor. During sick leave patients were able to plan the day based on personal needs and wishes. After returning to work, they had to adapt to a predetermined schedule. Recovery was considered to take a long time even if the process was progressing well.

“Take some of our patients. The wound is healed, the food intake is no problem, their bowel function is fine and they manage to take long walks. Yet, when these patients visit us three months after surgery, it becomes clear that they did not return to their pre-surgery level”.

#### Support and Encouragement

During hospitalization support came mainly from staff members, who worked to create optimal condition for recovery and encourage patient participation. The importance of listening and being sensitive to individual needs as well as motivating and helping patients use their own resources and capacity was stressed. After discharge a shift occurred in which encouragement and support now came chiefly from family, friends, and colleagues. It was reported that staff had limited experiences regarding the late phase of postoperative recovery. “I believe postoperative recovery includes much more than the things we experience.” Furthermore, it was described that many patients have no contact with the hospital or the primary health care center after being discharged. Having the possibility of maintaining contact with health care providers through telephone calls and visits to the primary care center was considered important to patients` inner sense of security after discharge.

The recovery process took place in different environments. The hospital was described as a safe setting where support and help were readily available. In hospital the patients were allowed to identify themselves as being ill, which made it easier to get the peace and quiet needed for recovery. Some informants reported that the hospital environment was adjusted to ill persons based on the design of, for example, the beds and toilets. However, others, who found the beds uncomfortable and thus inappropriate for sick persons, contradicted this statement. The hospital was also described as a strange environment with various odors and lighting. It was depicted as a place where it could be difficult to relax because one was constantly surrounded by unknown and ill persons. “It is not particular uplifting when everyone you see has an ailment or disease”. It was reported that the recovery process was normally faster in the home environment if there was some form of help and support, which, however, was not always the case.

#### Regular Appropriate Information

Adequate information, given according to individual needs and the person’s ability to understand, was thought to be of the utmost importance for successful postoperative recovery. The informants also pointed out the importance of receiving information at the right time.

“When one is affected by drugs, it is difficult to think clearly. I believe I would have had quite different questions and reflected about the situation differently if I were given a few more days to recover”.

The informants also pointed out the need to have information repeated. To obtain information about what is planned and then experience that the plans was actually carried out was seen as positive and crucial to the recovery process. Accounts of negative experiences included receiving misleading information or contradictory information from different sources. The possibility of obtaining information from other patients with similar experiences was stressed, and participants also noted that information could be acquired from the Internet.

#### Setbacks During Recovery

Setbacks, such as complications and unexpected events, delayed the recovery process. Some examples of complications were bleedings, wound infections, intestinal paralysis, and pneumonia but also included injuries caused by the surgery itself (e.g., neurological damage resulting in permanent impairment). The fact that the surgical procedure turned out to be more extensive than expected was interpreted as being very frustrating and a major setback. “They had opened me twice as much as I had originally expected. It was a great shock for me to discover that the stitches went all the way up to my sternum.” Suffering from another illness or disorder during the recovery period was also reported as a reversal, even if it was not linked directly to the surgery.

## DISCUSSION

This study reports on the overall recovery of patients in the current climate of shorter hospital stay following surgery. The study findings contribute to an increased understanding of postoperative recovery by emphasizing that it is a dynamic process during which individuals experiences subjective sensations whose existence and intensity vary over time. This increased understanding could contribute to a more personal care. Furthermore, the findings emphasizes that the recovery process is influenced by several factors unrelated to the type of surgery. This knowledge could increase the awareness among staff about the possibilities to affect the recovery process according to individual needs. The study findings elucidate the need not only to focus on surgery outcomes (such as length of hospitalization and the decrease of isolated symptoms) but also to have a broader overall perspective when studying postoperative recovery. Some of the findings, for example the return to normal everyday life focusing on specific symptoms [7, 9] and regaining functions [12], have been delineated earlier. Previous studies have focused mainly on short-term recovery after day surgery [24, 25], recovery during hospitalization [26], and recovery up to some weeks after discharge [27]. However, our findings demonstrate that the recovery process is extended over a longer period. During this period patients are mainly responsibly for their own recovery.

The recovery process constituted a path starting immediately after surgery and extending until the patient achieved independence in the activities of daily life and returned to everyday life. This is in accordance with the surgery trajectory discussed by Lawler [28] as demonstrated by turning points or recovery indicators. A question that should bed addressed is whether it is possible to define a standard achieved condition on an individual or group level after the recovery process has ended. Patients that, for example, have had a hip or knee replacement hope to achieve a physical function that is far better than before surgery. On the other hand, there might be situations where the patient will never return to the preoperative initial condition. There is great individual variation regarding level of independence or dependence in what we have called everyday life. In line with this reasoning, it is difficult to define an end point for recovery after surgery.

Our findings demonstrate that the postoperative recovery path was influenced by several factors unrelated to the type of surgery. In a previous study Wagner and colleagues [29] demonstrated that patients had quite different approaches to and strategies for dealing with recovery after hysterectomy. They suggested that this complicates the idea of having standardized plans for giving advice and guidance. Regarding further development of postoperative care, it is necessary to be familiar with the overall recovery process and integrate this with the knowledge of procedure-specific recovery needs. It has been suggested that a multidisciplinary collaboration between surgeons, nurses, anesthesiologists, and physiotherapists is a prerequisite to achieving improvements in perioperative care [30]. Yet, the major part of the recovery process takes place after leaving the hospital [3, 6] implying that the responsibility for recovery has been shifted from health professionals to the patients and their families. If patients are expected to be active in their own recovery, rather than relinquishing control to professional caregivers, there is a need for ongoing support during the recovery process, both at the hospital and after discharge. In the study referred to above of women’s experiences of short hospital stays after an abdominal hysterectomy, Wagner and colleagues reported that these women managed their physical situation well but needed the opportunity to communicate with staff after discharge. The importance of appropriate information on postoperative recovery has been underlined in several studies [17, 31, 32]. However, our findings indicate that staff has only limited insight into the things patients experience during the recovery process after discharge. Without appropriate knowledge of the patient’s needs and wishes after leaving hospital, it is difficult to give adequate support. We consider it the responsibility of the members of the health care team to inform, treat, support, and understand surgical patients during their recovery after surgery.

Besides the experiences of the core of the recovery, the knowledge generated from this study demonstrates that there are several ways of influencing the postoperative recovery process, such as support and encouragement from staff and family, being given the time to recover, and receiving regular appropriate information. This knowledge could be used to outline strategies for support and follow-up activities during postoperative recovery.

Our study agrees with a concept analysis stating that postoperative recovery is an energy-requiring process of returning to normality, including physiological, psychological, and social recovery as well as a return to previous habits [33]. The similarities between these findings concern, for example, the existence and complexity of different dimensions of postoperative recovery. However, this study provides additional knowledge by including a longterm perspective, showing that the recovery process is extended over a long time during which patients have to take care of their own recovery. Furthermore, it describes more than the core of recovery by elucidating that the recovery process is exposed to a variety of influencing factors. These two studies complement each other in their effort to increase our knowledge and understanding of postoperative recovery.

The findings from this study could also be understood in relation to Levine’s [34] conservation model, which focuses on individuals as holistic beings and identifies adaptation as the process by which the wholeness, or the integrity, of the individual is maintained. Four conservation principles – conservation of energy, personal integrity, structural integrity, and social integrity – are facilitating the adaptation process. The unpleasant physical symptoms and disturbances in emotional well-being described in this study (e.g., fatigue, pain, anxiety, and depression) as well as the regaining of functions and reestablishment of activities could be assumed to have effect on energy and personal, structural, and social integrity. Together these factors are related to a return to wholeness. The conservation principles could be used, singly or together, to identify interventions with the goal of maintaining the person’s wholeness during the postoperative recovery process.

To acquire a wide variety of experiences, we viewed it as necessary to take the subjective experiences of patients and the professional knowledge of various health care personnel into account. Consequently, we decided to combine staff and patient interviews, even though it resulted in a large amount of data. The choice of method offered good possibilities to describe the experiences of patients and staff with regard to postoperative recovery. There is a lack of studies examining postoperative recovery from a longterm perspective. In an effort to obtain a broad description of postoperative recovery, including both a time perspective and experiences, we decided to use a wide time range, between 3 weeks and 1 year after surgery, in the individual interviews. This seems to be a long time interval, and the question could be raised as to whether it is possible to remember experiences several months later. However, the participants were able to describe their experiences during the recovery process in detail. To undergo surgery and recover from it seemed to be a strong experience. The participants that had a longer time perspective appeared to have reflected on their situation compared to those who had undergone surgery more recently. The patients who participated in one of the focus group interviews were still hospitalized. The fact that it was close to the surgery, within just a few days of postoperative experiences, might have affected their capacity to interact with each other. This group was more focused on telling their own individual stories than sharing and discussing their experiences.

To address how the findings could be transferred to other groups or contexts, we have provided a thorough description of the selection and characteristics of participants, data collection, and analysis. To help ensure trustworthiness, participants with various experiences and perspectives were chosen [23]. Our claim is that this study design has provided a straight summary description [18] of postoperative recovery. Finally, we believe that this understanding of the recovery process could be of value for caregivers in a variety of health care systems, such as postanesthesia care units, surgical wards, and primary health care settings in their efforts to support their patients during recovery after surgery.

## CONCLUSION

We have learned from this study that postoperative recovery is a dynamic process extended over a period when individuals experience a variety of subjective sensations. Putting this knowledge into practice is a prerequisite for discharge planning and support of the ongoing recovery process at home. These findings elucidate the need to have a broad, overall perspective when studying postoperative recovery. However, it also raises new questions. We are at present conducting a study with the purpose of identifying the recovery process after different surgical procedures.

## Figures and Tables

**Fig. (1):**
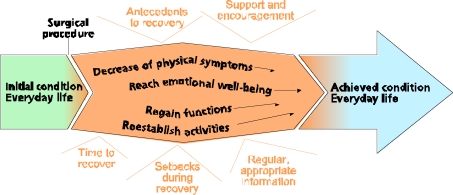
The postoperative recovery process.
